# Association between FGFR2 (rs2981582, rs2420946 and rs2981578) polymorphism and breast cancer susceptibility: a meta-analysis

**DOI:** 10.18632/oncotarget.13839

**Published:** 2016-12-09

**Authors:** Yafei Zhang, Xianling Zeng, Pengdi Liu, Ruofeng Hong, Hongwei Lu, Hong Ji, Le Lu, Yiming Li

**Affiliations:** ^1^ Department of General Surgery, The Second Affiliated Hospital of Xi'an Jiaotong University, Xi'an, Shaanxi 710004, China; ^2^ Department of Obstetrics and Gynecology, The First Affiliated Hospital of Xi'an Jiaotong University, Xi’an, Shaanxi 710061, China

**Keywords:** breast cancer, FGFR2, polymorphism

## Abstract

The association between fibroblast growth factor receptor 2 (FGFR2) polymorphism and breast cancer (BC) susceptibility remains inconclusive. The purpose of this systematic review was to evaluate the relationship between FGFR2 (rs2981582, rs2420946 and rs2981578) polymorphism and BC risk. PubMed, Web of science and the Cochrane Library databases were searched before October 11, 2015 to identify relevant studies. Odds ratios (ORs) and 95% confidence intervals (CIs) were used to estimate the strength of associations. Sensitivity and subgroup analyses were conducted. Thirty-five studies published from 2007 to 2015 were included in this meta-analysis. The pooled results showed that there was significant association between all the 3 variants and BC risk in any genetic model. Subgroup analysis was performed on rs2981582 and rs2420946 by ethnicity and Source of controls, the effects remained in Asians, Caucasians, population-based and hospital-based groups. We did not carryout subgroup analysis on rs2981578 for the variant included only 3 articles. This meta-analysis of case-control studies provides strong evidence that FGFR2 (rs2981582, rs2420946 and rs2981578) polymorphisms were significantly associated with the BC risk. For rs2981582 and rs2420946, the association remained significant in Asians, Caucasians, general populations and hospital populations. However, further large scale multicenter epidemiological studies are warranted to confirm this finding and the molecular mechanism for the association need to be elucidated further.

## INTRODUCTION

Breast cancer (BC), one of the most common malignant tumors among women worldwide, has the highest mortality rate in female cancer. Its incidence rate is increasing year by year and the patients are becoming younger and younger in the world [1, 2]. BC is the result of the interaction of environmental and genetic factors. Under the same carcinogenic factors, only a small fraction of people develop BC, which suggests that the genetic background differences lead to individual differences in BC susceptibility [3].

In recent years, genome-wide association study (GWAS) provides a good technical support for the study on the susceptibility loci with high variation frequency and low penetrance [4]. Large numbers of BC related susceptibility genes and single nucleotide polymorphism sites have been found through GWAS, such as LSP1, MAP3K1, FGFR2, TGFB1, TOX3, etc [5]. The discovery of these genes will have an important impact on the prevention and treatment of BC, especially FGFR2 (rs2981582, rs2420946 and rs2981578). FGFR2 gene is located in 10q26, and contains at least 22 exons [6]. FGFR2 is a member of the tyrosine kinase receptor family. It is a transmembrane protein, and is mainly composed of three parts: extracellular region, transmembrane region and intracellular region. The extracellular segment has three immunoglobulin like protein functional areas. Through the combination with FGFs, the functional areas could activate the tyrosine kinase activity and induce receptor tyrosine phosphorylation. It also starts series of cascade reaction through the RAS-MAPK, JAK-STATs and PLC-Y signal system, and then regulate the transcription of downstream genes involve in the body's physiological and pathological activities, such as cell proliferation, differentiation, migration and apoptosis, angiogenesis, skeletal development. So FGFR2 plays an important role in the processes of human growth and development [7].

Lots of researches have reported the association between FGFR2 (rs2981582, rs2420946 and rs2981578) polymorphism and BC risk. However, due to differences in ethnic and regional and other factors, the conclusions of related reports are still inconclusive. Raskin et al [8] found FGFR2 rs2420946 was significantly associated with BC risk in Ashkenazi and Sephardi Jews, with a similar but not significant trend in Arabs. Liang et al's [9] study indicated that each of thesingle nucleotide polymorphisms (SNPs) (rs2981582and rs2420946) was significantly associated with increased BC risk, and the risk was the highest for those carrying the 2 mutation sites at the same time. While, there are also some different reports. Liu et al [10] found that FGFR2 rs2420946was not significantly correlated with the occurrence of BC in Chinese population. These different conclusions may result from the diversity of genetic background and carcinogenic factors, therefore, further studies in different populations should be implemented to assess the correlation between SNPs and BC risk. Although five meta-analysis [11–15] on the associations between FGFR2 (rs2981582, rs2420946 and rs2981578) polymorphism and BC risk had been implemented, yet the results remained inconclusive and some just no subgroup. Therefore, we carried out this meta-analysis on all the included case-control researches to make a more accurate assessment of the relationship.

## RESULTS

### Characteristics of included papers

**Figure 1:**
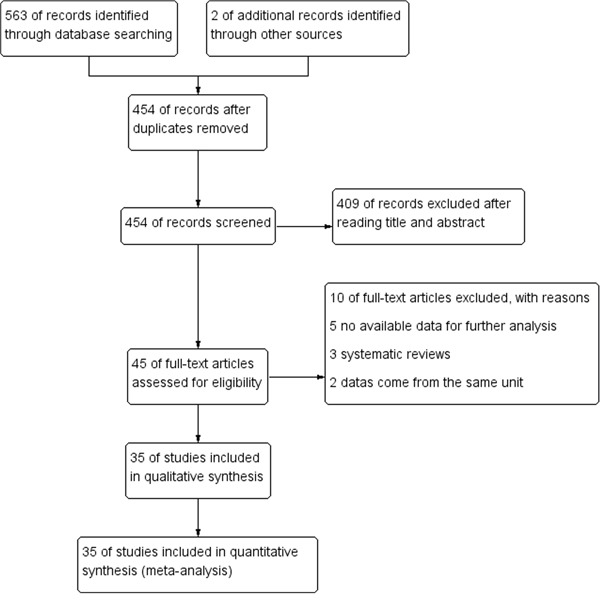
Flow chart of studies selection in this meta-analysis

The specific search process is shown in Figure 1. A total of 563 references were preliminarily identified at first based on our selection strategy. We also identified 2 papers through other sources. 454 records left after removing repeated studies. We refer to titles or abstracts of all the included literatures, and then removed obviously irrelevant papers. In the end, the whole of the rest of the papers were checked based on the inclusion and exclusion criteria. Finally, 35 studies on FGFR2 (rs2981582, rs2420946 and rs2981578) polymorphism and the occurrence of BC were eventually included in our study. Characteristics of eligible analysis are shown in Table 1. The 35 case-control papers were published between 2007 and 2015, among them, 1 study was performed in African, 17 in Asian, 14 in Caucasians and 3 in both Asian and Caucasians. All studies were case-controlled.

**Table 1:** Characteristics of the studies included in the meta-analysis

First author	Year	Country	Ethnicity	Source of controls	Genotyping medthod	Number(case/control)	HWE
rs2981582 (C>T)							
Kawase [20]	2009	Japan	Asian	HB	TaqMan	455/912	0.773315
Hu [25]	2011	China	Asian	PB	PCR-RFLP	203/200	0.758366
Li [26]	2011	China	Asian	HB	MassArray	401/441	0.219207
Chen [27]	2012	China	Asian	PB	PCR-SSCP	388/424	0.048991
Butt [28]	2012	Swedish	Caucasian	PB	MassArray	713/1399	0.816442
Shan [29]	2012	Tunisian	African	PB	TaqMan	600/358	0.060883
Fu [30]	2012	China	Asian	HB	iPLEX	118/104	0.474243
Campa [31]	2011	Mixed	Mixed	PB	Taqman	8313/11594	0.607558
Slattery [32]	2011	American	Caucasian	PB	Taqman	1734/2040	0.822253
Han [33]	2011	Korean	Asian	PB	Taqman	3232/3489	0.361342
Tamimi [34]	2010	Swedish	Caucasian	PB	Taqman	680/734	0.535243
Gorodnova [35]	2010	Russian	Caucasian	NA	Taqman	140/174	0.000621
Ren [36]	2010	China	Asian	HB	Taqman	956/471	0.024883
McInerney [37]	2009	British	Caucasian	PB	KASPar	941/997	0.83057
Boyarskikh [38]	2009	Russia	Caucasian	PB	Taqman	744/628	0.659988
Garcia-Closas [39]	2008	Mixed	Mixed	PB, HB	Taqman	16882/26058	0.892667
Liang [9]	2008	China	Asian	HB	Taqman	1026/1062	0.97418
Antoniou [40]	2008	European	Mixed	NA	Taqman, MALDI-TOF	4990/4301	0.596563
Zhao [41]	2010	China	Asian	HB	PCR-RFLP	956/471	0.024883
Xi [42]	2014	China	Asian	HB	MALDI-TOF	815/849	0.959015
Campa [19]	2015	Mixed	Caucasian	PB	TaqMan	1234/12231	0.779613
Slattery [43]	2013	American	Caucasian	PB	multiplexed bead array	3560/4138	0.364662
Chan [44]	2012	China	Asian	HB	Taqman	1168/1475	0.164674
Dai [45]	2012	China	Asian	HB	TaqMan	1768/1844	0.423521
Jara [46]	2013	Chile	Caucasian	PB	TaqMan	351/802	0.138274
Liang [18]	2015	China	Asian	HB	MassARRAY	607/856	0.298476
Liu [47]	2013	China	Asian	HB	PCR-RFLP	203/200	0.758366
Murillo-Zamora [48]	2013	Mexico	Caucasian	PB	Multiplexed assays	687/907	0.351295
Ottini [49]	2013	Italy	Caucasian	PB	TaqMan	413/745	0.76716
Ozgoz [50]	2013	Turkey	Caucasian	PB	PCR-RFLP	31/30	0.281979
Siddiqui [51]	2014	India	Asian	HB	PCR-RFLP	368/484	0.526174
rs2420946 (C>T)							
Raskin [8]	2008	USA	Caucasian	PB	TaqMan	1480/1474	0.224235
Kawase [20]	2009	Japan	Asian	HB	TaqMan	453/912	0.519554
Liu [10]	2009	China	Asian	PB	PCR-RFLP	106/116	0.361602
Hu [25]	2011	China	Asian	PB	PCR-RFLP	203/200	0.325727
Li [26]	2011	China	Asian	HB	MassArray	391/432	0.703117
Fu [30]	2012	China	Asian	HB	iPLEX	118/104	0.505449
Liang [9]	2008	China	Asian	HB	Taqman	1020/1050	0.413194
Hunter [52]	2007	USA	Caucasian	PB	Taqman	2912/3212	0.293864
Jara [46]	2013	Chile	Caucasian	PB	TaqMan	351/802	0.292806
Liang [18]	2015	China	Asian	HB	MassARRAY	603/847	0.063645
Liu [47]	2013	China	Asian	HB	PCR-RFLP	203/200	0.325727
rs2981578 (A>G)							
Chen [27]	2012	China	Asian	PB	PCR-SSCP	378/458	0.290218
Lin [53]	2012	Taiwan	Asian	PB	PCR-RFLP	87/70	0.724138
Siddiqui [51]	2014	India	Asian	HB	PCR-RFLP	368/484	0.278456

HWE: hardy-weinberg equilibrium; PB: population based; HB: hospital-based.

### Meta-analysis results

**Figure 2:**
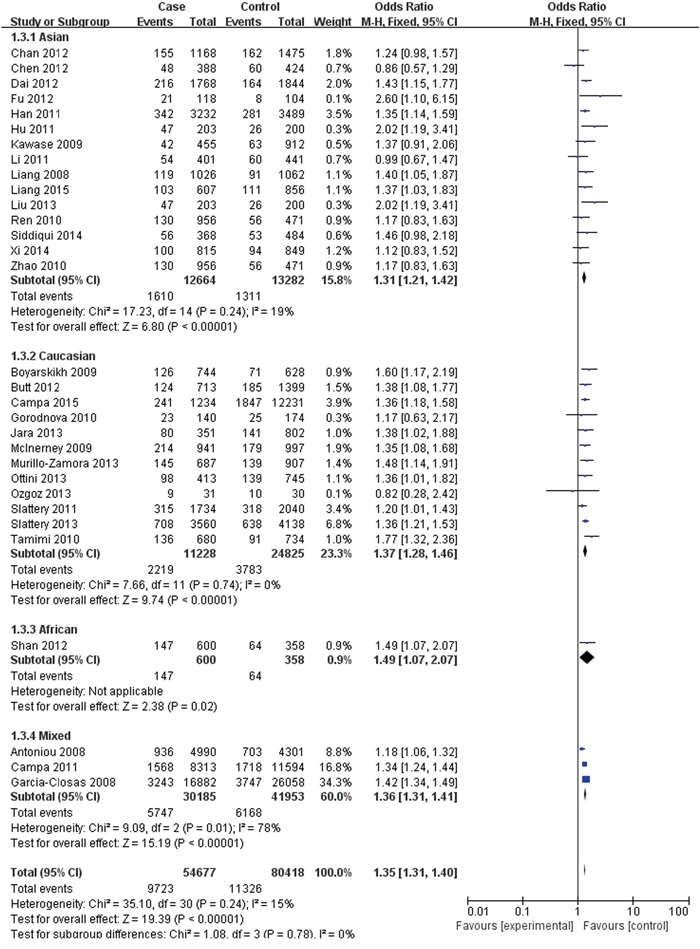
Forest plots of rs2981582 (C>T) polymorphism and breast cancer risk stratified by ethnicity (Recessive model TT vs. CC + TC)

**Figure 3:**
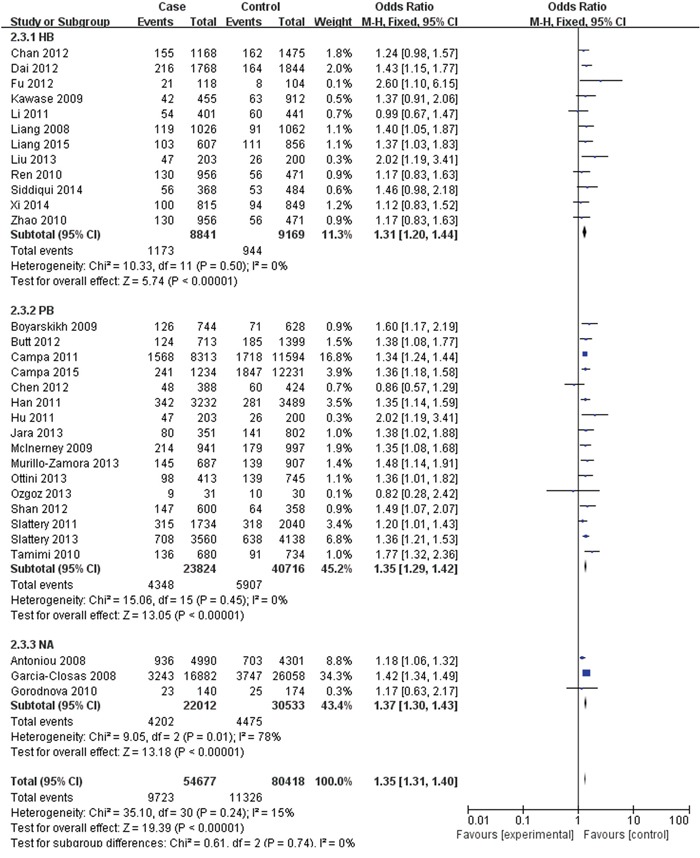
Forest plots of rs2981582 (C>T) polymorphism and breast cancer risk stratified by Source of controls (Recessive model TT vs. CC + TC)

The FGFR2 (rs2981582, rs2420946 and rs2981578) polymorphisms genotype distribution and allele frequencies incase groups and control groups were shown in Table 2. Main results of our study were shown in Table 3. There were 31 studies with 54,677 cases and 80,418 controls for FGFR2 rs2981582 variants. As shown in Table 3, Figure 2 and Figure 3, the pooled results indicated that the correlation between FGFR2 rs2981582 polymorphism and the occurrence of BC was significant in any genetic model: Allele model (OR: 1.23; 95% CI: 1.19- 1.26; P< 0.00001), Dominant model (OR: 1.29; 95% CI: 1.24-1.34; P< 0.00001), Recessive model (OR: 1.35; 95% CI: 1.31-1.40; P<0.00001), Homozygous genetic model (OR: 1.50; 95% CI: 1.42-1.58; P< 0.00001), Heterozygote comparison (OR: 1.22; 95% CI: 1.17-1.27; P< 0.00001). In ethnicity specific analysis, FGFR2 rs2981582 were significantly associated with BC risk both in Asians (Allele model: OR=1.19, 95% CI=1.15- 1.24, P< 0.00001; Dominant model: OR=1.23, 95% CI=1.17-1.29, P< 0.00001; Recessive model: OR=1.31, 95% CI=1.21-1.42, P< 0.00001; Homozygous genetic model: OR=1.42, 95% CI=1.31-1.54, P< 0.00001; Heterozygote comparison: OR=1.18, 95% CI=1.12-1.25, P< 0.00001) and Caucasians (Allele model: OR=1.25, 95% CI=1.21-1.30, P< 0.00001; Dominant model: OR=1.33, 95% CI=1.26-1.40, P< 0.00001; Recessive model: OR=1.37, 95% CI=1.28-1.46, P< 0.00001; Homozygous genetic model: OR=1.56, 95% CI=1.45-1.68, P< 0.00001; Heterozygote comparison: OR=1.26, 95% CI=1.19-1.33, P< 0.00001). We didn't discuss the African subgroup for just one study from African. The analysis in different source of controls showed the same association between FGFR2 rs2981582 polymorphism and BC susceptibility both in HB(Allele model: OR=1.22, 95% CI=1.16-1.27, P< 0.00001; Dominant model: OR=1.27, 95% CI=1.20-1.35, P< 0.00001; Recessive model: OR=1.31, 95% CI=1.20-1.44, P< 0.00001; Homozygous genetic model: OR=1.45, 95% CI=1.31-1.60, P< 0.00001; Heterozygote comparison: OR=1.23, 95% CI=1.15-1.31, P< 0.00001) and PB(Allele model: OR=1.24, 95% CI=1.19-1.29, P< 0.00001; Dominant model: OR=1.31, 95% CI=1.23-1.40, P< 0.00001; Recessive model: OR=1.35, 95% CI=1.29-1.42, P< 0.00001; Homozygous genetic model: OR=1.50, 95% CI=1.43-1.58, P< 0.00001; Heterozygote comparison: OR=1.23, 95% CI=1.15-1.31, P< 0.00001).

**Table 2:** Polymorphisms genotype distribution and allele frequency in cases and controls

First author	Genotype (N)								Allele frequency (N)			
	Case				Control				Case		Control	
rs2981582 (C>T)	Total	TT	TC	CC	Total	TT	TC	CC	T	C	T	C
Kawase [20]	455	42	192	221	912	63	347	502	276	634	473	1351
Hu [25]	203	47	78	78	200	26	95	79	172	234	147	253
Li [26]	401	54	180	167	441	60	189	192	288	514	309	573
Chen [27]	388	48	208	132	424	60	224	140	304	472	344	504
Butt [28]	713	124	356	233	1399	185	653	561	604	822	1023	1775
Shan [29]	600	147	315	138	358	64	154	140	609	591	282	434
Fu [30]	118	21	55	42	104	8	47	49	97	139	63	145
Campa [31]	8313	1568	3951	2794	11594	1718	5456	4420	7087	9539	8892	14296
Slattery [32]	1734	315	884	535	2040	318	981	741	1514	1954	1617	2463
Han [33]	3232	342	1393	1497	3489	281	1457	1751	2077	4387	2019	4959
Tamimi [34]	680	136	304	240	734	91	324	319	576	784	506	962
Gorodnova [35]	140	23	67	50	174	25	54	95	113	167	104	244
Ren [36]	956	130	400	426	471	56	181	234	660	1252	293	649
McInerney [37]	941	214	458	269	997	179	483	335	886	996	841	1153
Boyarskikh [38]	744	126	371	247	628	71	273	284	623	865	415	841
Garcia-Closas [39]	16882	3243	8218	5421	26058	3747	12255	10056	14704	19060	19749	32367
Liang [9]	1026	119	460	447	1062	91	439	532	698	1354	621	1503
Antoniou [40]	4990	936	2407	1647	4301	703	2051	1547	4279	5701	3457	5145
Zhao [41]	956	130	400	426	471	56	181	234	660	1252	293	649
Xi [42]	815	100	423	292	849	94	376	379	623	1007	564	1134
Campa [19]	1234	241	608	385	12231	1847	5793	4591	1090	1378	9487	14975
Slattery [43]	3560	708	1749	1103	4138	638	2009	1491	3165	3955	3285	4991
Chan [44]	1168	155	527	486	1475	162	618	695	837	1499	942	2008
Dai [45]	1768	216	820	732	1844	164	796	884	1252	2284	1124	2564
Jara [46]	351	80	178	93	802	141	366	295	338	364	648	956
Liang [18]	607	103	266	238	856	111	375	370	472	742	597	1115
Liu [47]	203	47	78	78	200	26	95	79	172	234	147	253
Murillo-Zamora [48]	687	145	309	233	907	139	415	353	599	775	693	1121
Ottini [49]	413	98	205	110	745	139	361	245	401	425	639	851
Ozgoz [50]	31	9	16	6	30	10	12	8	34	28	32	28
Siddiqui [51]	368	56	168	144	484	53	205	226	280	456	311	657
rs2420946 (C>T)	Total	TT	TC	CC	Total	TT	TC	CC	T	C	T	C
Raskin [8]	1480	356	715	409	1474	285	700	489	1427	1533	1270	1678
Kawase [20]	453	60	226	167	912	99	416	397	346	560	614	1210
Liu [10]	106	16	51	39	116	21	51	44	83	129	93	139
Hu [25]	203	50	92	61	200	34	105	61	192	214	173	227
Li [26]	391	74	186	131	432	68	202	162	334	448	338	526
Fu [30]	118	25	55	38	104	9	48	47	105	131	66	142
Liang [9]	1020	163	519	338	1050	142	505	403	845	1195	789	1311
Hunter [52]	2912	603	1409	900	3212	484	1562	1166	2615	3209	2530	3894
Jara [46]	351	85	175	91	802	143	374	285	345	357	660	944
Liang [18]	603	116	297	190	847	145	379	323	529	677	669	1025
Liu [47]	203	50	92	61	200	34	105	61	192	214	173	227
rs2981578 (A>G)	Total	GG	GA	AA	Total	GG	GA	AA	G	A	G	A
Chen [27]	378	150	188	40	458	160	212	86	488	268	532	384
Lin [53]	87	35	39	13	70	21	36	13	109	65	78	62
Siddiqui [51]	368	129	185	54	484	151	228	105	443	293	530	438

**Table 3:** Meta-analysis results

Outcome or Subgroup	Studies	Participants	Statistical Method	Effect Estimate	P value	Heterogeneity	
						I^2^	P value
Allele model							
rs2981582 (C>T)	31	270190	OR (M-H, Random, 95% CI)	1.23 [1.19, 1.26]	< 0.00001	41%	0.01
Asian	15	51892	OR (M-H, Fixed, 95% CI)	1.19 [1.15, 1.24]	< 0.00001	0%	0.54
Caucasian	12	72106	OR (M-H, Fixed, 95% CI)	1.25 [1.21, 1.30]	< 0.00001	4%	0.4
HB	12	36020	OR (M-H, Fixed, 95% CI)	1.22 [1.16, 1.27]	< 0.00001	0%	0.87
PB	16	129080	OR (M-H, Random, 95% CI)	1.24 [1.19, 1.29]	< 0.00001	46%	0.02
rs2420946 (C>T)	11	34378	OR (M-H, Fixed, 95% CI)	1.23 [1.18, 1.29]	< 0.00001	0%	0.67
Asian	8	13916	OR (M-H, Fixed, 95% CI)	1.19 [1.11, 1.28]	< 0.00001	0%	0.67
Caucasian	3	20462	OR (M-H, Fixed, 95% CI)	1.26 [1.19, 1.33]	< 0.00001	0%	0.53
HB	6	12666	OR (M-H, Fixed, 95% CI)	1.20 [1.12, 1.29]	< 0.00001	0%	0.61
PB	5	21712	OR (M-H, Fixed, 95% CI)	1.25 [1.18, 1.32]	< 0.00001	0%	0.5
rs2981578 (A>G)	3	3690	OR (M-H, Fixed, 95% CI)	1.29 [1.13, 1.47]	0.0002	0%	0.93
Dominant model							
rs2981582 (C>T)	31	135095	OR (M-H, Random, 95% CI)	1.29 [1.24, 1.34]	< 0.00001	46%	0.003
Asian	15	25946	OR (M-H, Fixed, 95% CI)	1.23 [1.17, 1.29]	< 0.00001	0%	0.63
Caucasian	12	36053	OR (M-H, Fixed, 95% CI)	1.33 [1.26, 1.40]	< 0.00001	16%	0.28
HB	12	18010	OR (M-H, Fixed, 95% CI)	1.27 [1.20, 1.35]	< 0.00001	0%	0.89
PB	16	64540	OR (M-H, Random, 95% CI)	1.31 [1.23, 1.40]	< 0.00001	55%	0.004
rs2420946 (C>T)	11	17189	OR (M-H, Fixed, 95% CI)	1.28 [1.20, 1.37]	< 0.00001	0%	0.77
Asian	8	6958	OR (M-H, Fixed, 95% CI)	1.25 [1.13, 1.39]	< 0.00001	0%	0.75
Caucasian	3	10231	OR (M-H, Fixed, 95% CI)	1.31 [1.20, 1.42]	< 0.00001	0%	0.38
HB	6	6333	OR (M-H, Fixed, 95% CI)	1.28 [1.15, 1.42]	< 0.00001	0%	0.73
PB	5	10856	OR (M-H, Fixed, 95% CI)	1.29 [1.19, 1.40]	< 0.00001	0%	0.44
rs2981578 (A>G)	3	1845	OR (M-H, Fixed, 95% CI)	1.71 [1.32, 2.21]	< 0.0001	0%	0.63
Recessive model							
rs2981582 (C>T)	31	135095	OR (M-H, Fixed, 95% CI)	1.35 [1.31, 1.40]	< 0.00001	15%	0.24
Asian	15	25946	OR (M-H, Fixed, 95% CI)	1.31 [1.21, 1.42]	< 0.00001	19%	0.24
Caucasian	12	36053	OR (M-H, Fixed, 95% CI)	1.37 [1.28, 1.46]	< 0.00001	0%	0.74
HB	12	18010	OR (M-H, Fixed, 95% CI)	1.31 [1.20, 1.44]	< 0.00001	0%	0.5
PB	16	64540	OR (M-H, Fixed, 95% CI)	1.35 [1.29, 1.42]	< 0.00001	0%	0.45
rs2420946 (C>T)	11	17189	OR (M-H, Fixed, 95% CI)	1.36 [1.26, 1.48]	< 0.00001	4%	0.4
Asian	8	6958	OR (M-H, Fixed, 95% CI)	1.27 [1.12, 1.45]	0.0003	8%	0.37
Caucasian	3	10231	OR (M-H, Fixed, 95% CI)	1.42 [1.29, 1.57]	< 0.00001	0%	0.61
HB	6	6333	OR (M-H, Fixed, 95% CI)	1.27 [1.11, 1.46]	0.0006	4%	0.39
PB	5	10856	OR (M-H, Fixed, 95% CI)	1.41 [1.28, 1.56]	< 0.00001	0%	0.46
rs2981578 (A>G)	3	1845	OR (M-H, Fixed, 95% CI)	1.24 [1.02, 1.50]	0.03	0%	0.75
Homozygous genetic model							
rs2981582 (C>T)	31	71786	OR (M-H, Random, 95% CI)	1.50 [1.42, 1.58]	< 0.00001	33%	0.04
Asian	15	14673	OR (M-H, Fixed, 95% CI)	1.42 [1.31, 1.54]	< 0.00001	2%	0.43
Caucasian	12	18824	OR (M-H, Fixed, 95% CI)	1.56 [1.45, 1.68]	< 0.00001	0%	0.73
HB	12	10192	OR (M-H, Fixed, 95% CI)	1.45 [1.31, 1.60]	< 0.00001	0%	0.69
PB	16	34101	OR (M-H, Fixed, 95% CI)	1.50 [1.43, 1.58]	< 0.00001	32%	0.11
rs2420946 (C>T)	11	8925	OR (M-H, Fixed, 95% CI)	1.52 [1.39, 1.66]	< 0.00001	0%	0.54
Asian	8	3629	OR (M-H, Fixed, 95% CI)	1.40 [1.21, 1.62]	< 0.00001	0%	0.57
Caucasian	3	5296	OR (M-H, Fixed, 95% CI)	1.60 [1.43, 1.79]	< 0.00001	0%	0.56
HB	6	3303	OR (M-H, Fixed, 95% CI)	1.43 [1.22, 1.66]	< 0.00001	0%	0.53
PB	5	5622	OR (M-H, Fixed, 95% CI)	1.57 [1.41, 1.76]	< 0.00001	0%	0.47
rs2981578 (A>G)	3	957	OR (M-H, Fixed, 95% CI)	1.80 [1.36, 2.39]	< 0.0001	0%	0.8
Heterozygote genetic model							
rs2981582 (C>T)	31	114046	OR (M-H, Random, 95% CI)	1.22 [1.17, 1.27]	< 0.00001	42%	0.007
Asian	15	23025	OR (M-H, Fixed, 95% CI)	1.18 [1.12, 1.25]	< 0.00001	1%	0.44
Caucasian	12	30051	OR (M-H, Fixed, 95% CI)	1.26 [1.19, 1.33]	< 0.00001	26%	0.19
HB	12	15893	OR (M-H, Fixed, 95% CI)	1.23 [1.15, 1.31]	< 0.00001	0%	0.75
PB	16	54285	OR (M-H, Random, 95% CI)	1.23 [1.15, 1.31]	< 0.00001	52%	0.009
rs2420946 (C>T)	11	14127	OR (M-H, Fixed, 95% CI)	1.21 [1.13, 1.29]	< 0.00001	0%	0.69
Asian	8	5852	OR (M-H, Fixed, 95% CI)	1.21 [1.08, 1.34]	0.0005	0%	0.62
Caucasian	3	8275	OR (M-H, Fixed, 95% CI)	1.21 [1.11, 1.32]	< 0.0001	0%	0.37
HB	6	5348	OR (M-H, Fixed, 95% CI)	1.23 [1.10, 1.38]	0.0002	0%	0.66
PB	5	8779	OR (M-H, Fixed, 95% CI)	1.19 [1.09, 1.30]	< 0.0001	0%	0.42
rs2981578 (A>G)	3	1199	OR (M-H, Fixed, 95% CI)	1.65 [1.26, 2.16]	0.0003	0%	0.51

CI: Confidence interval.

**Figure 4:**
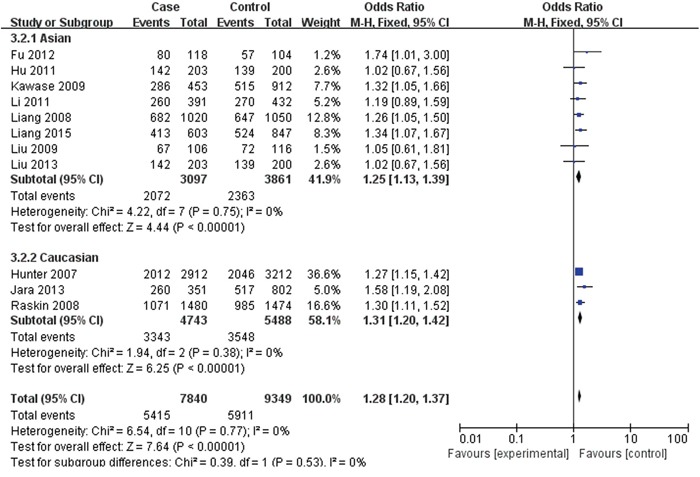
Forest plots of rs2420946 (C>T) polymorphism and breast cancer risk stratified by ethnicity (Dominant model TC + TT vs. CC)

**Figure 5:**
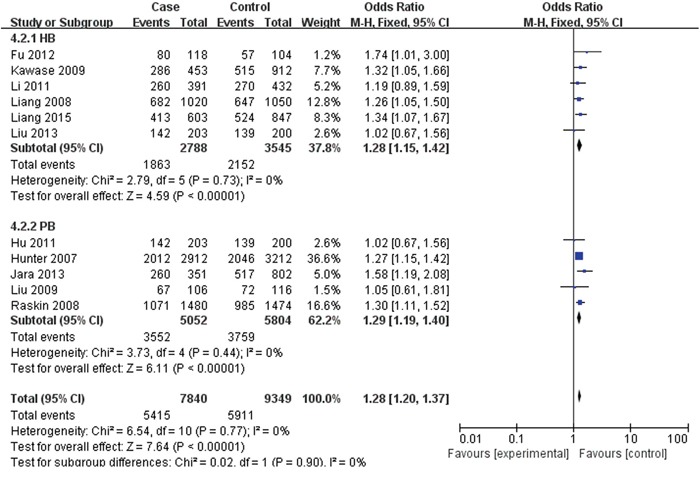
Forest plots of rs2420946 (C>T) polymorphism and breast cancer risk stratified by Source of controls (Dominant model TC + TT vs. CC)

For rs2420946, 11 studies with 7,840 cases and 9,349 controls were included to assess the association. As shown in Table 3, Figure 4 and Figure 5, the pooled ORs suggested that rs2420946 was significantly associated with BC susceptibility in all the five genetic models: Allele model 1.23 (95% CI: 1.18-1.29; P< 0.00001), Dominant model 1.28 (95% CI: 1.20-1.37; P< 0.00001), Recessive model 1.36 (95% CI: 1.26-1.48; P< 0.00001), Homozygous genetic model 1.52 (95% CI: 1.39-1.66; P< 0.00001), Heterozygote comparison 1.21 (95% CI: 1.13-1.29; P< 0.00001). When stratified by Ethnicity and Source of controls, the results showed that FGFR2 rs2420946 was significantly associated with BC risk in Asians, Caucasians, HB and PB.

**Figure 6:**
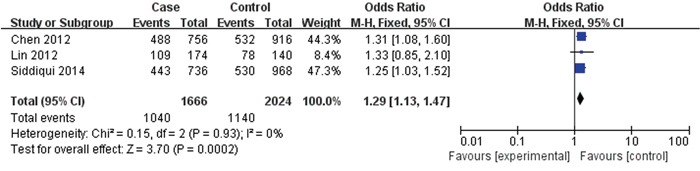
Forest plots of rs2981578 (A>G) polymorphism and breast cancer risk (Allele model G vs. A)

3 papers with 833 cases and 1012 controls were adopted to evaluate the association between the rs2981578 polymorphism and the BC risk. As shown in Table 3, Figure 6, the association between rs2981578 variant and BC susceptibility was also significant in any genetic model (Allele model: OR= 1.29, 95% CI= 1.13-1.47, P= 0.0002; Dominant model: OR= 1.71, 95% CI= 1.32-2.21, P< 0.0001; Recessive model: OR= 1.24, 95% CI= 1.02-1.50, P= 0.03; Homozygous genetic model: OR= 1.80, 95% CI= 1.36-2.39, P< 0.0001; Heterozygote comparison: OR= 1.65, 95% CI= 1.26-2.16, P= 0.0003).

### Sensitivity analyses

As shown in Table 1, all the studies conformed to the balance of HWE in controls except Chen’s(2012), Gorodnova’s(2012), Ren’s(2012), Zhao’s(2012) studies(P<0.05) in rs2981582 group, however, after performing the sensitivity analyses, the overall outcomes were no statistically significant change when removing any of the articles, indicating that our study has good stability and reliability.

### Detection for heterogeneity

Heterogeneity among studies was obtained by *Q* statistic. Random-effect models were applied if *p*-value of heterogeneity tests were less than 0.1 (*p* ≤ 0.1), otherwise, fixed-effect models were selected (Table 3).

### Publication bias

**Figure 7:**
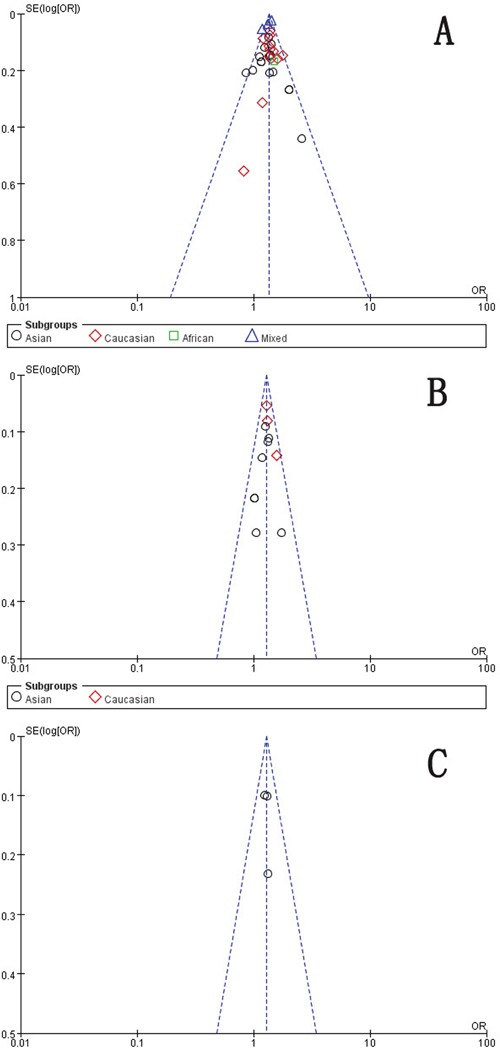
Funnel plot assessing evidence of publication bias **A**. rs2981582 (C>T) (Recessive model TT vs. CC + TC). **B**. rs2420946 (C>T) (Dominant model TC + TT vs. CC). **C**. rs2981578 (A>G) (Allele model G vs. A). SE: standard error; OR: odds ratio.

As Figure 7 indicated, the symmetrical funnel plot indicated that there is no significant publication bias in the total population. We use Begg's funnel plot and Egger test to evaluate the published bias, no significant publication bias was found in the Begg's test and Egger's test (P>0.05).

## DISCUSSION

FGFR2 has been proved to be associated with many diseases, especially the relationship between FGFR2 and cancer, which has become a hot research topic in recent years [16]. GWAS analysis revealed that FGFR2 gene was one of the BC susceptibility genes. There are 8 SNPs(rs35054928, rs2981578, rs2912778, rs2912781, rs35393331, rsl0736303, rs7895676, rs33971856) in its second intron and the SNPs of FGFR2 have become the hotspot in BC susceptibility gene study [17–19]. But the difference of SNPs allele frequency and LD structure reflects the difference of the genetic variation in the race, so the occurrence and characteristics of BC were different. Therefore, a variation in one study does not have the same risk impact on other crowds. This requires repeated studies on previously related locis in multiple populations worldwide.

Lots of researches have reported the association between FGFR2 (rs2981582, rs2420946 and rs2981578) polymorphism and BC risk. However, due to differences in ethnic and regional and other factors, the conclusions of related reports are still inconclusive. Thus, we conducted the meta-analysis to evaluate the relationship between FGFR2 (rs2981582, rs2420946 and rs2981578) polymorphism and BC risk.

In our study, there were 31 studies with 54,677 cases and 80,418 controls for FGFR2 rs2981582 variants. In the total population, the pooled results indicated that the correlation between FGFR2 rs2981582 polymorphism and the occurrence of BC was significant in any genetic model. Furthermore, in ethnicity-specific analysis, FGFR2 rs2981582 were also significantly associated with BC risk both in Asians and Caucasians. We didn't discuss the African subgroup for just one study was from African. The analysis in different source of controls showed the same association between FGFR2 rs2981582 polymorphism and BC susceptibility both in HB and PB, indicating that both hospital populations and general populations followed the same relationship. For rs2420946, 11 studies with 7,840 cases and 9,349 controls were included to assess the association. In the total population, the pooled ORs suggested that rs2420946 was significantly associated with BC susceptibility in all the five genetic models. When stratified by ethnicity and source of controls, the results showed the same association in Asians, Caucasians, hospital populations and general populations, indicating that different genetic backgrounds and living environment were not strong enough to change these associations. All the results for the two variants (rs2981582, rs2420946) were partially consistent with the consequences of Wang's [13], Peng's [14], Zhang's [12] and Jia's [15] meta-analysis, while they didn't conduct analysis in different source of controls, making our results more valuable. Furthermore, they didn't use all the five genetic models(allele model, dominant model, recessive model, homozygous model and heterozygous model) to assess the strength of association. Wang's [13] study also reported that the association appeared to be much stronger for estrogen receptor-positive and progesterone receptor-positive BC, which was not analyzed in our study. Peng's [14] study was conducted on the base of present mata-ananlyses, which may missed some individual studies with larger sample sizes, and this type meta-analysis may not appropriate. In Zhang's [12] study, the increased risk was found in the subgroup of postmenopausal women for rs2420946. However, only one study [20] reported that risk in premenopausal women. For Jia's [15] study, in the ethnicity subgroup, using Non-Caucasians represent different ethnicities may cause some heterogeneity.

Three articles with 833 cases and 1012 controls were adopted to evaluate the association between the rs2981578 polymorphism and the BC risk. As the preceding two variants, the association between rs2981578 variant and BC susceptibility was also significant in any genetic model. For just only 3 studies, no stratified study was conducted for rs2981578 polymorphism. However, in Zhou's [11] meta-analysis, they found that rs2981578 polymorphism might decrease BC risk. This may result from the literature selection bias. While the sample size of our study for rs2981578 was so small, data from a large sample of multiple centers is still needed to assess the association.

Our meta-analysis has several limitations. First, our study is a summary of the data. For lack of all individual raw data, we could not assess the cancer risk stratified by other covariates including age, sex, environment, hormone level, menopause age and other risk factors. We also cannot analyze the potential interaction of gene-environment and gene-gene. Second, only published papers were included in our meta-analysis, there still may be some unpublished studies which are in line with the conditions. Therefore, publication bias may exist even no statistical evidence was found in the meta-analysis. Third, for just only 3 papers, no stratified study was conducted for rs2981578 polymorphism. Moreover, our study is a summary of the data. We did not verify it from the level of molecular mechanism. Data from large scale multicenter epidemiological studies is still needed to confirm the relationship between FGFR2 (rs2981582, rs2420946 and rs2981578) polymorphisms and BC risk, and the molecular mechanism for the associations need to be elucidated further.

In conclusion, our meta-analysis based on case-control studies provides strong evidence that FGFR2 (rs2981582, rs2420946 and rs2981578) polymorphisms are significantly associated with the BC risk. For rs2981582 and rs2420946, the association remained significant in Asians, Caucasians, general populations and hospital populations. However, further large scale multicenter epidemiological studies are warranted to confirm this finding and the molecular mechanism for the associations need to be elucidated further.

## MATERIALS AND METHODS

### Literature search

We searched PubMed, Web of science and the Cochrane Library for relevant studies published before October 11, 2015. The following keywords were used: (Fibroblast Growth Factor Receptor 2 or FGFR2) and (variant* or genotype or polymorphism or SNP) and (breast) and (cancer or carcinom* or neoplasm* or tumor), and the combined phrases for all genetic studies on the association between the FGFR2 (rs2981582, rs2420946 and rs2981578) polymorphism and BC risk. The reference lists of all articles were also manually screened for potential studies. Abstracts and citations were screened by two researchers independently. All the eligible articles need a second screening for the full-text. The searching was done without language limitations.

### Selection and exclusion criteria

Inclusion criteria: A study was included in this meta-analysis if it met the following criteria: *i*)independent case-control studies for humans; *ii*) the study evaluating the association between FGFR2 (rs2981582, rs2420946 and rs2981578) polymorphism and BC risk; *iii*) the study presenting available genotype frequencies in cancer cases and control subjects for risk estimated; *iiii*) cases should have been diagnosed by a pathological examination. We excluded comments, editorials, systematic reviews and studies lacking sufficient data or studies with male cases. If the researches were duplicated or shared in more than one study, the most recent publications were included.

### Data extraction and synthesis

We used endnote bibliographic software to construct an electronic library of citations identified in the literature search. All the PubMed, Web of science and the Cochrane Library searches were performed using Endnote. Duplicates were found automatically by endnote and deleted manually. All data extraction were checked and calculated twice according to the inclusion criteria listed above by two independent investigators. Data extracted from the included studies were as follows: First author, year of publication, country, Ethnicity, Source of controls, Genotyping method, number of cases and controls and evidence of Hardy-Weinberg equilibrium(HWE) in controls. A third reviewer would participate if some disagreements were emerged, and a final decision was made by the majority of the votes.

### Statistical analysis

All statistical analyses were performed using STATA version 11.0 software (StataCorp LP, College Station, TX) and Review Manage version 5.2.0 (The Cochrane Collaboration, 2012). Hardy-Weinberg equilibrium (HWE) was assessed by *χ^2^* test in the control group of each study [21]. The strength of associations between the FGFR2 (rs2981582, rs2420946 and rs2981578) polymorphism and BC risk were measured by odds ratio (ORs) with 95% confidence interval (CIs). *Z* test was used to assess the significance of the ORs, *I^2^* and *Q* statistics was used to determine the statistical heterogeneity among studies. A random-effect model was used if *p* value of heterogeneity tests was no more than 0.1 (*p* ≤ 0.1), and otherwise, a fixed-effect model was selected [21, 22]. Sensitivity analyses were performed to assess the stability of the results. We used Begg's funnel plot and Egger's test to evaluate the publication bias [23, 24]. The strength of the association was estimated in the allele model, the dominant model, the recessive model, the homozygous genetic model and the heterozygous genetic model, respectively. *p*< 0.05 was considered statistically significant. We performed subgroup according to Ethnicity and Source of controls.
